# The genome sequence of the red fox, *Vulpes vulpes *(Linnaeus, 1758)

**DOI:** 10.12688/wellcomeopenres.23516.1

**Published:** 2025-01-14

**Authors:** Rosa Lopez Colom, Michelle O’Brien, Natural History Museum Genome Acquisition Lab, Darwin Tree of Life Barcoding collective, Wellcome Sanger Institute Tree of Life Management, Samples and Laboratory team, Wellcome Sanger Institute Scientific Operations: Sequencing Operations, Wellcome Sanger Institute Tree of Life Core Informatics team, Tree of Life Core Informatics collective, Darwin Tree of Life Consortium

**Affiliations:** 1 Wildfowl & Wetlands Trust, Slimbridge, Gloucestershire, England, UK

**Keywords:** Vulpes vulpes, red fox, genome sequence, chromosomal, Carnivora

## Abstract

We present a genome assembly from an individual female *Vulpes vulpes* (red fox; Chordata, Mammalia, Carnivora, Canidae). The assembly comprises two haplotypes, with total lengths of 2,411.71 megabases and 2,398.53 megabases, respectively. For both haplotypes, 97.8% of haplotype 1 and 97.97% of haplotype 2 are scaffolded into 17 chromosomal pseudomolecules. Additionally, the mitochondrial genome has been assembled, with a total length of 16.68 kilobases.

## Species taxonomy

Eukaryota; Opisthokonta; Metazoa; Eumetazoa; Bilateria; Deuterostomia; Chordata; Craniata; Vertebrata; Gnathostomata; Teleostomi; Euteleostomi; Sarcopterygii; Dipnotetrapodomorpha; Tetrapoda; Amniota; Mammalia; Theria; Eutheria; Boreoeutheria; Laurasiatheria; Carnivora; Caniformia; Canidae; *Vulpes*; *Vulpes vulpes* (Linnaeus, 1758) (NCBI:txid)

## Background

The red fox ( *Vulpes vulpes*) is the largest species within its genus, exhibiting subtle sexual dimorphism. Males typically weigh between 5.5 and 9 kg, while females, or vixens, weigh between 3.5 and 7.5 kg. This canid species is characterised by a distinctive red coat with a white undertone that extends from the chin, through the chest, and along the abdomen. The tail is heavily furred, also red in colour with a white tip, and the ears are black-tipped ( Couper, 2016).

Red foxes are found across a wide range of habitats throughout the Palaearctic region, including woodlands, wetlands, deserts, shrublands, and urban areas. They were also historically introduced to North America, Australia, and various British Overseas Territories and Crown Dependencies, with varying degrees of success in population establishment and impact on local ecosystems ( Hoffmann & Sillero-Zubiri, 2021).

The breeding season for red foxes extends from January to March, with the first cubs emerging from their dens at around one month of age in April. In urban environments, casualties among cubs and vixens are common during this time as cubs begin to practise survival skills through play, and mothers move their cubs to different locations if they sense a threat ( Smith, 2016), leading to increased road traffic incidents. Additionally, well-meaning members of the public sometimes mistakenly assume that cubs have been abandoned, leading to further risks.

Although the red fox is classified as a species of least concern by the IUCN Red List, its role as a meso-predator and its impact on conservation projects make it a species of significant ecological interest. Meso-predator management is important not only in rural and countryside habitats but also in urban areas, where the interactions between foxes and domestic animals, such as cats, influence local ecology and prey-predator dynamics ( Molsher *et al.*, 2017).

Genomic analysis of red foxes would provide valuable insights into their interactions with other species and their effects on prey populations, relevant to species recovery conservation projects. It would also inform public health strategies, wildlife management, and help address human-wildlife conflicts.

## Genome sequence report

**Figure 1.**
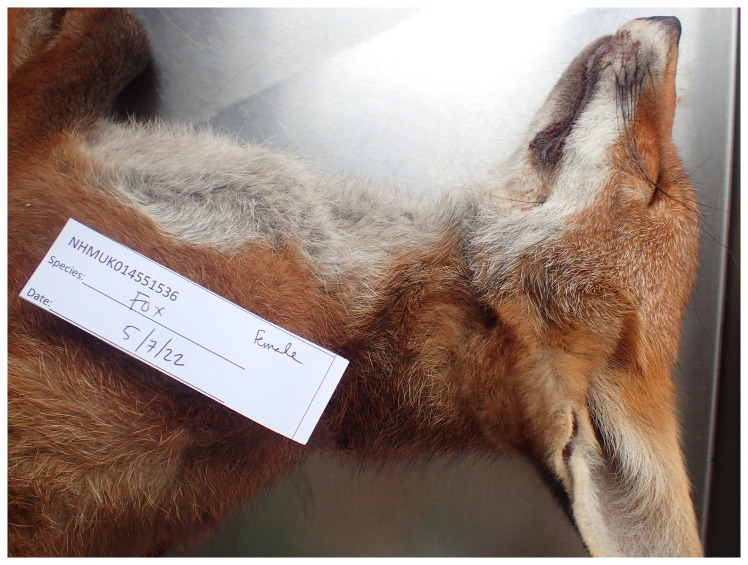
Photograph of the *Vulpes vulpes* (mVulVul1) specimen from which muscle samples were taken for genome sequencing.

The genome of an adult female *Vulpes vulpes* ( Figure 1) was sequenced using Pacific Biosciences single-molecule HiFi long reads, generating a total of 67.99 Gb (gigabases) from 5.88 million reads, providing approximately 28-fold coverage. Primary assembly contigs were scaffolded with chromosome conformation Hi-C data, which produced 324.41 Gb from 2,148.40 million reads. Specimen and sequencing details are provided in Table 1.

**Table 1.** Specimen and sequencing data for *Vulpes vulpes*.

Project information			
**Study title**	Vulpes vulpes (red fox)		
**Umbrella BioProject**	PRJEB74591		
**Species**	*Vulpes vulpes*		
**BioSample**	SAMEA113398840		
**NCBI taxonomy ID**	9627		
Specimen information			
**Technology**	**ToLID**	**BioSample ** **accession**	**Organism part**
**PacBio long read sequencing**	mVulVul1	SAMEA113398901	muscle
**Hi-C sequencing**	mVulVul1	SAMEA113398901	muscle
**RNA sequencing**	mVulVul1	SAMEA113398901	muscle
Sequencing information			
**Platform**	**Run accession**	**Read count**	**Base count (Gb)**
**Illumina NovaSeq X (Hi-C)**	ERR12862084	2.15e+09	324.41
**Revio (PacBio)**	ERR12875147	5.88e+06	67.99
**Illumina NovaSeq X (RNA)**	ERR13493935	1.17e+08	17.63

**Figure 2.**
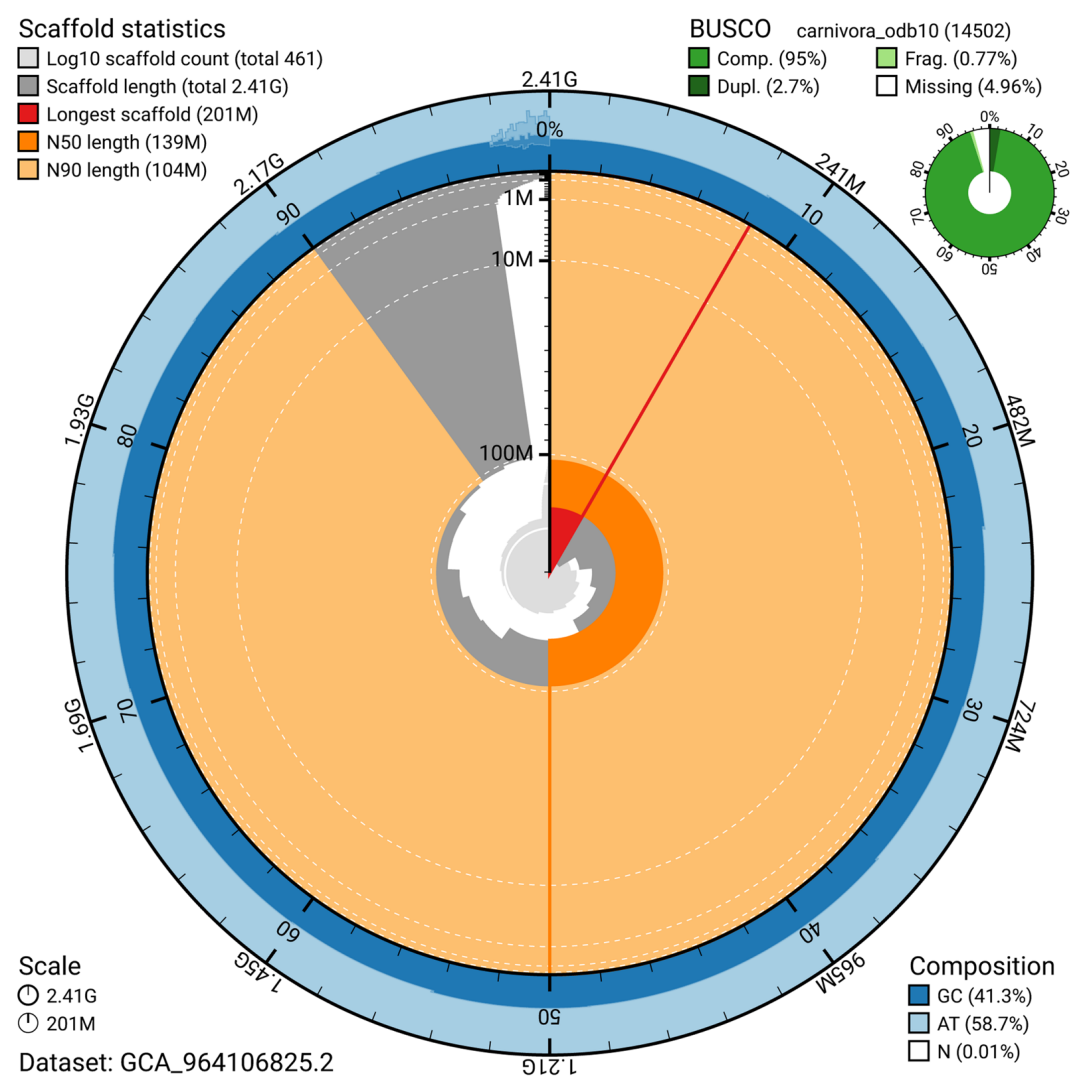
Genome assembly of *Vulpes vulpes*, mVulVul1.hap1.2: metrics. The BlobToolKit snail plot provides an overview of assembly metrics and BUSCO gene completeness. The circumference represents the length of the whole genome sequence, and the main plot is divided into 1,000 bins around the circumference. The outermost blue tracks display the distribution of GC, AT, and N percentages across the bins. Scaffolds are arranged clockwise from longest to shortest and are depicted in dark grey. The longest scaffold is indicated by the red arc, and the deeper orange and pale orange arcs represent the N50 and N90 lengths. A light grey spiral at the centre shows the cumulative scaffold count on a logarithmic scale. A summary of complete, fragmented, duplicated, and missing BUSCO genes in the carnivora_odb10 set is presented at the top right. An interactive version of this figure is available at https://blobtoolkit.genomehubs.org/view/Vulpes_vulpes/dataset/GCA_964106825.2/snail.

**Figure 3.**
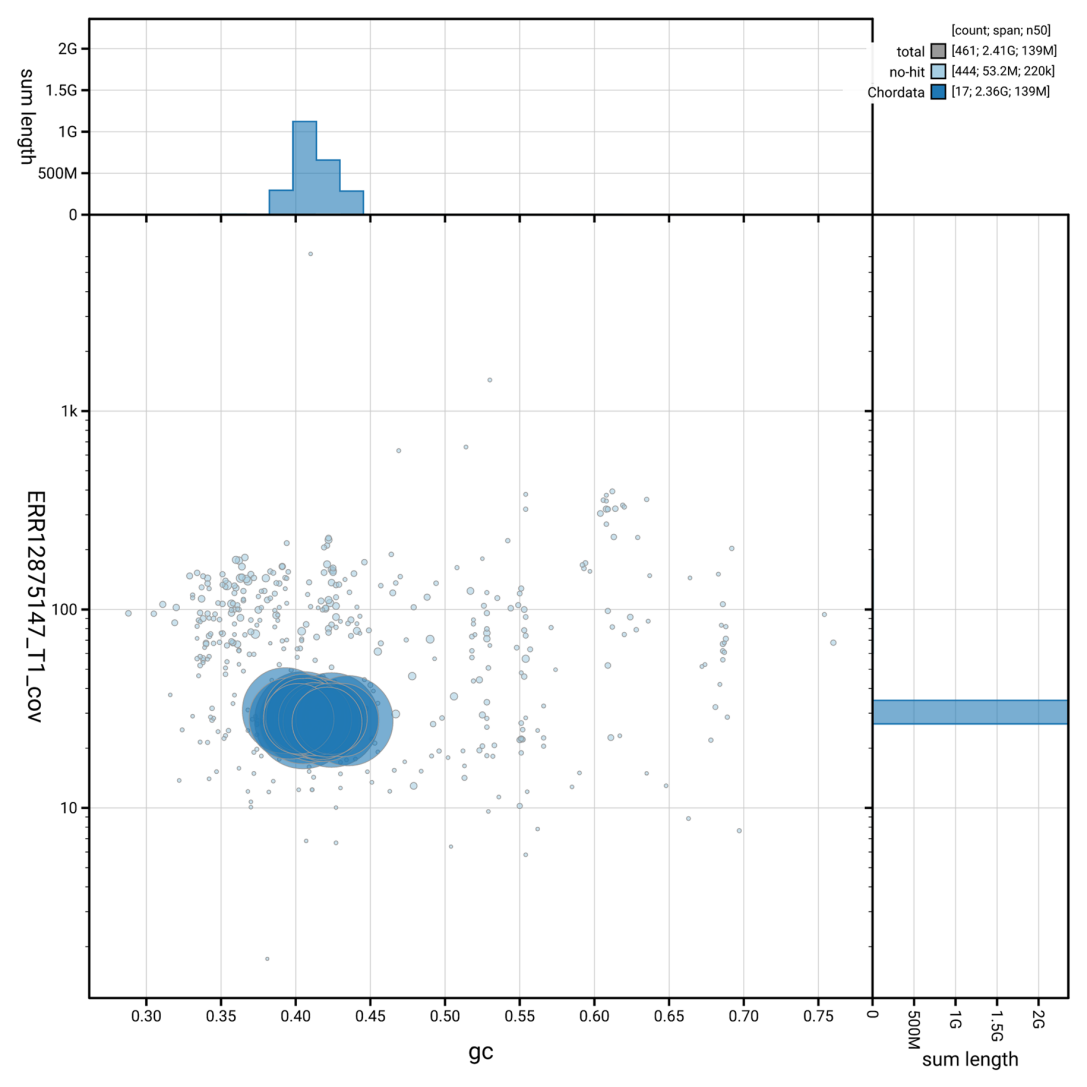
Genome assembly of *Vulpes vulpes*, mVulVul1.hap1.2: BlobToolKit GC-coverage plot. Blob plot showing sequence coverage (vertical axis) and GC content (horizontal axis). The circles represent scaffolds, with the size proportional to scaffold length and the colour representing phylum membership. The histograms along the axes display the total length of sequences distributed across different levels of coverage and GC content. An interactive version of this figure is available at https://blobtoolkit.genomehubs.org/view/Vulpes_vulpes/dataset/GCA_964106825.2/blob.

**Figure 4.**
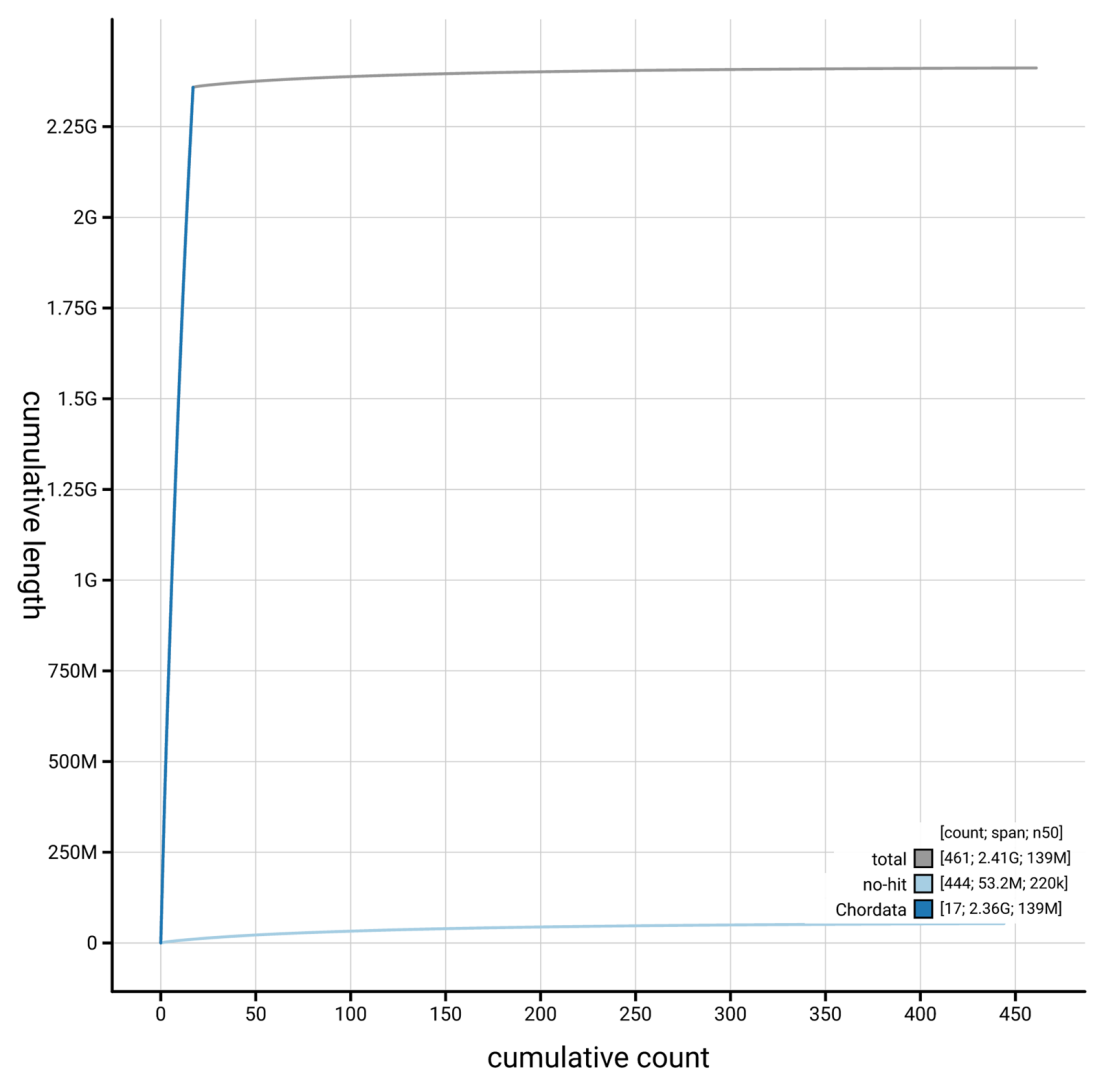
Genome assembly of *Vulpes vulpes* mVulVul1.hap1.2: BlobToolKit cumulative sequence plot. The grey line shows cumulative length for all scaffolds. Coloured lines show cumulative lengths of scaffolds assigned to each phylum using the buscogenes taxrule. An interactive version of this figure is available at https://blobtoolkit.genomehubs.org/view/Vulpes_vulpes/dataset/GCA_964106825.2/cumulative.

The two haplotypes were combined for curation. Manual assembly curation corrected 87 missing joins or mis-joins. This reduced the scaffold number by 4.75%. The final haplotype 1 assembly has a total length of 2,411.71 Mb in 460 sequence scaffolds, with 1,845 gaps, and a scaffold N50 of 139.24 Mb ( Table 2). The snail plot in Figure 2 provides a summary of the assembly statistics, while the distribution of assembly scaffolds on GC proportion and coverage is shown in Figure 3. The cumulative assembly plot in Figure 4 shows curves for subsets of scaffolds assigned to different phyla.

**Table 2.** Genome assembly data for *Vulpes vulpes*, mVulVul1.hap1.2.

Genome assembly	Haplotype 1	Haplotype 2
Assembly name	mVulVul1.hap1.2	mVulVul1.hap2.2
Assembly accession	GCA_964106825.2	GCA_964106925.2
Assembly level	chromosome	chromosome
Span (Mb)	2,411.71	2,398.53
Number of contigs	2,305	2,169
Number of scaffolds	460	350
Longest scaffold (Mb)	200.87	197.92
Assembly metrics *	Haplotype 1	Haplotype 2
Contig N50 length (≥ 1 Mb)	2.0 Mb	2.1 Mb
Scaffold N50 length (= chromosome N50)	139.2 Mb	139.5 Mb
Consensus quality (QV) (≥ 40)	62.3	62.4
*k*-mer completeness	92.00%	91.68%
Combined *k*-mer completeness (≥ 95%)	99.65%	
BUSCO ** (S > 90%; D < 5%)	C:95.1%[S:92.4%,D:2.7%], F:0.8%,M:4.1%,n:14,502	C:94.6%[S:92.0%,D:2.6%], F:0.8%,M:4.6%,n:14,502
Percentage of assembly mapped to chromosomes (≥ 90%)	97.8%	97.97%
Sex chromosomes (localised homologous pairs)	X	X
Organelles (one complete allele)	Mitochondrial genome: 16.68 kb	

* Assembly metric benchmarks are adapted from Rhie *et al.* (2021) and the Earth BioGenome Project Report on Assembly Standards September 2024. ** BUSCO scores based on the carnivora_odb10 BUSCO set using version 5.4.3. C = complete [S = single copy, D = duplicated], F = fragmented, M = missing, n = number of orthologues in comparison.

**Figure 5.**
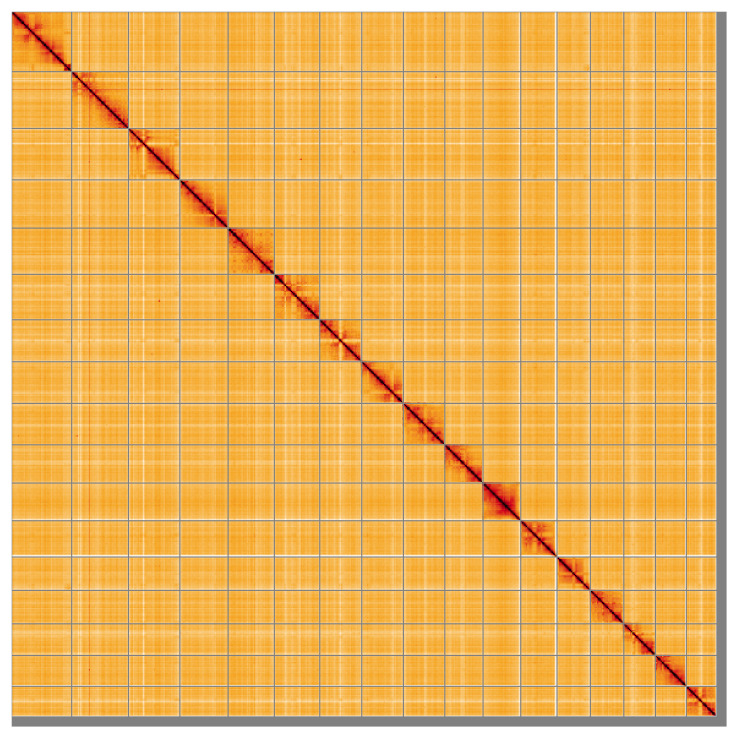
Genome assembly of *Vulpes vulpes* mVulVul1.hap1.2: Hi-C contact map of the mVulVul1.hap1.2 assembly, visualised using HiGlass. Chromosomes are shown in order of size from left to right and top to bottom. An interactive version of this figure may be viewed at https://genome-note-higlass.tol.sanger.ac.uk/l/?d=U5ZyziwHRtSJQTz5wJUC6g.

Most (97.8%) of the assembly sequence was assigned to 17 chromosomal-level scaffolds, representing 16 autosomes and the X chromosome. Chromosome-scale scaffolds confirmed by the Hi-C data are named in order of size ( Figure 5; Table 3). This genome has been assembled using PacBio and Hi-C data and phased. The result is two curated haplotypes. Chromosome X was assigned by Hi-C signal. The mitochondrial genome was also assembled and is included both as a contig within the multifasta file of the genome submission and as a standalone record in GenBank.

**Table 3.** Chromosomal pseudomolecules in the genome assembly of *Vulpes vulpes*, mVulVul1.

Haplotype 1				Haplotype 2			
INSDC accession	Name	Length (Mb)	GC%	INSDC accession	Name	Length (Mb)	GC%
**OZ067313.1**	1	200.87	40.5	**OZ067162.1**	1	197.92	40.5
**OZ067314.1**	2	189.89	42.5	**OZ067163.1**	2	190.33	42.5
**OZ067315.1**	3	172.19	43.5	**OZ067164.1**	3	171.43	43.5
**OZ067316.1**	4	160.91	40.5	**OZ067165.1**	4	160.51	40.5
**OZ067317.1**	5	155.1	39.5	**OZ067166.1**	5	153.31	39.5
**OZ067318.1**	6	151.96	41.5	**OZ067167.1**	6	152.11	41.5
**OZ067319.1**	7	139.75	41.5	**OZ067168.1**	7	140.02	41.5
**OZ067320.1**	8	139.24	42	**OZ067169.1**	8	139.45	42
**OZ067321.1**	9	139.11	39.5	**OZ067170.1**	9	137.61	39.5
**OZ067322.1**	10	127.72	40.5	**OZ067171.1**	10	127.04	40.5
**OZ067324.1**	11	120.98	41.5	**OZ067173.1**	11	121.84	41.5
**OZ067325.1**	12	111.93	43	**OZ067175.1**	12	111.15	43
**OZ067326.1**	13	111.74	41.5	**OZ067174.1**	13	111.93	41.5
**OZ067327.1**	14	106.05	42.5	**OZ067176.1**	14	104.34	42.5
**OZ067328.1**	15	103.55	40	**OZ067177.1**	15	103.81	40
**OZ067329.1**	16	101.58	42	**OZ067178.1**	16	102.02	42
**OZ067323.1**	X	125.95	40.5	**OZ067172.1**	X	125.03	40.5
**OZ067330.1**	MT	0.02	41				

The estimated Quality Value (QV) of the final haplotype 1 assembly is 62.3 with *k*-mer completeness of 92.0%, and the assembly has a BUSCO v5.4.3 completeness of 95.1% (single = 92.4%, duplicated = 2.7%), using the carnivora_odb10 reference set ( *n* = 14,502).

For haplotype 2, the estimated Quality Value (QV) of the final assembly is 62.4 with *k*-mer completeness of 91.68%. The assembly has a BUSCO v5.4.3 completeness of 94.6% (single = 92.0%, duplicated = 2.6%), using the carnivora_odb10 reference set ( *n* = 14,502).

## Methods

### Sample acquisition and DNA barcoding

Several small samples of posterior thigh muscles were collected from a deceased female red fox, *Vulpes vulpes* (specimen ID NHMUK014551536, ToLID mVulVul1). This Canidae species was collected in Newgounds, Gloucester, England, United Kingdom (latitude 51.73, longitude–2.40) on 2022-07-05 after a road traffic incident. The specimen was identified by Rosa Lopez Colom (Wildfowl & Wetlands Trust) and preserved by freezing (–20 °C).

The initial identification was verified by an additional DNA barcoding process according to the framework developed by Twyford *et al*. (2024). A small sample was dissected from the specimens and stored in ethanol, while the remaining parts were shipped on dry ice to the Wellcome Sanger Institute (WSI). The tissue was lysed, the COI marker region was amplified by PCR, and amplicons were sequenced and compared to the BOLD database, confirming the species identification ( Crowley *et al.*, 2023). Following whole genome sequence generation, the relevant DNA barcode region was also used alongside the initial barcoding data for sample tracking at the WSI ( Twyford *et al.*, 2024). The standard operating procedures for Darwin Tree of Life barcoding have been deposited on protocols.io ( Beasley *et al.*, 2023).

### Nucleic acid extraction

The workflow for high molecular weight (HMW) DNA extraction at the Wellcome Sanger Institute (WSI) Tree of Life Core Laboratory includes a sequence of core procedures: sample preparation and homogenisation, DNA extraction, fragmentation and purification. Detailed protocols are available on protocols.io ( Denton *et al.*, 2023). The mVulVul1 sample was prepared for DNA extraction by weighing and dissecting it on dry ice ( Jay *et al.*, 2023). Tissue from the muscle was cryogenically disrupted using the Covaris cryoPREP ^®^ Automated Dry Pulverizer ( Narváez-Gómez *et al.*, 2023). HMW DNA was extracted using the Automated MagAttract v2 protocol ( Oatley *et al.*, 2023a). DNA was sheared into an average fragment size of 12–20 kb in a Megaruptor 3 system ( Bates *et al.*, 2023). Sheared DNA was purified by solid-phase reversible immobilisation, using AMPure PB beads to eliminate shorter fragments and concentrate the DNA ( Oatley *et al.*, 2023b). The concentration of the sheared and purified DNA was assessed using a Nanodrop spectrophotometer and Qubit Fluorometer using the Qubit dsDNA High Sensitivity Assay kit. Fragment size distribution was evaluated by running the sample on the FemtoPulse system.

RNA was extracted from muscle tissue of mVulVul1 in the Tree of Life Laboratory at the WSI using the RNA Extraction: Automated MagMax™ *mir*Vana protocol ( do Amaral *et al.*, 2023). The RNA concentration was assessed using a Nanodrop spectrophotometer and a Qubit Fluorometer using the Qubit RNA Broad-Range Assay kit. Analysis of the integrity of the RNA was done using the Agilent RNA 6000 Pico Kit and Eukaryotic Total RNA assay. RNA data is publicly available and will be used for gene annotation.

### Hi-C preparation

Tissue from the muscle of the mVulVul1 sample was processed at the WSI Scientific Operations core, using the Arima-HiC v2 kit. Frozen tissue (stored at –80 °C) was fixed, and the DNA crosslinked using a TC buffer with 22% formaldehyde. After crosslinking, the tissue was homogenised using the Diagnocine Power Masher-II and BioMasher-II tubes and pestles. Following the kit manufacturer's instructions, crosslinked DNA was digested using a restriction enzyme master mix. The 5’-overhangs were then filled in and labelled with biotinylated nucleotides and proximally ligated. An overnight incubation was carried out for enzymes to digest remaining proteins and for crosslinks to reverse. Prior to library preparation, a clean up was performed with SPRIselect beads.

### Library preparation and sequencing

Library preparation and sequencing were performed by the WSI Scientific Operations core. 

***PacBio HiFi sequencing***

At the minimum, samples were required to have an average fragment size (previously mode fragment size) exceeding 8 kb and a total mass over 400 ng to proceed to the low input SMRTbell Prep Kit 3.0 protocol (Pacific Biosciences, California, USA).

Libraries were prepared using the SMRTbell Prep Kit 3.0 as per the manufacturer's instructions. The kit includes the reagents required for end repair/A-tailing, adapter ligation, post-ligation SMRTbell bead cleanup, and nuclease treatment. Following the manufacturer’s instructions, size selection and clean up was carried out using diluted AMPure PB beads (Pacific Biosciences, California, USA). DNA concentration was quantified using the Qubit Fluorometer v4.0 (Thermo Fisher Scientific) with Qubit 1X dsDNA HS assay kit and the final library fragment size analysis was carried out using the Agilent Femto Pulse Automated Pulsed Field CE Instrument (Agilent Technologies) and gDNA 55kb BAC analysis kit.

Prepared libraries were normalised to 2nM and 15μL used for making complexes. For libraries below 2nM all 10uL was used for making complexes. Primers were annealed and polymerases were hybridised to create circularised complexes according to manufacturer’s instructions. The complexes were purified with the 1.2X clean up with SMRTbell beads. The purified complexes were then diluted to the Revio loading concentration, between 200 -300pM, and spiked with a Revio sequencing internal control.

Samples were sequenced using the Revio system on Revio 25M SMRT cells. The SMRT link software, a PacBio web-based end-to-end workflow manager, was used to set-up and monitor the run, as well as perform primary and secondary analysis of the data upon completion.

***Hi-C library preparation and sequencing***

For Hi-C library preparation, DNA was fragmented to a size of 400 to 600 bp using a Covaris E220 sonicator. The DNA was then enriched, barcoded, and amplified using the NEBNext Ultra II DNA Library Prep Kit following manufacturers’ instructions. The Hi-C sequencing was performed using paired-end sequencing with a read length of 150 bp on an Illumina NovaSeq X instrument.

Poly(A) RNA-Seq libraries were constructed using the NEB Ultra II RNA Library Prep kit, following the manufacturer’s instructions. RNA sequencing was performed on the Illumina NovaSeq X instrument.

### Genome assembly, curation and evaluation

***Assembly***

Prior to assembly of the PacBio HiFi reads, a database of *k*-mer counts ( *k* = 31) was generated from the filtered reads using FastK. GenomeScope2 ( Ranallo-Benavidez *et al.*, 2020) was used to analyse the *k*-mer frequency distributions, providing estimates of genome size, heterozygosity, and repeat content.

The HiFi reads were first assembled using Hifiasm ( Cheng *et al.*, 2021; Cheng *et al.*, 2022) in Hi-C phasing mode, resulting in a pair of haplotype-resolved assemblies. The Hi-C reads were mapped to the primary contigs using bwa-mem2 ( Vasimuddin *et al.*, 2019). The contigs were further scaffolded using the provided Hi-C data ( Rao *et al.*, 2014) in YaHS ( Zhou *et al.*, 2023) using the --break option for handling potential misassemblies. The scaffolded assemblies were evaluated using Gfastats ( Formenti *et al.*, 2022), BUSCO ( Manni *et al.*, 2021) and Merqury.FK ( Rhie *et al.*, 2020).

The mitochondrial genome was assembled using MitoHiFi ( Uliano-Silva *et al.*, 2023), which runs MitoFinder ( Allio *et al.*, 2020) and uses these annotations to select the final mitochondrial contig and to ensure the general quality of the sequence.

***Assembly curation***

The assembly was decontaminated using the Assembly Screen for Cobionts and Contaminants (ASCC) pipeline (article in preparation). Flat files and maps used in curation were generated in TreeVal ( Pointon *et al.*, 2023). Manual curation was primarily conducted using PretextView ( Harry, 2022), with additional insights provided by JBrowse2 ( Diesh *et al.*, 2023) and HiGlass ( Kerpedjiev *et al.*, 2018). Scaffolds were visually inspected and corrected as described by Howe *et al*. (2021). Any identified contamination, missed joins, and mis-joins were corrected, and duplicate sequences were tagged and removed. The curation process is documented at https://gitlab.com/wtsi-grit/rapid-curation (article in preparation).

***Assembly quality assessment***

The Merqury.FK tool ( Rhie *et al.*, 2020) was used to evaluate *k*-mer completeness and assembly quality for the curated haplotypes (Hap1/2), using the *k*-mer databases ( *k* = 31) that were pre-computed prior to genome assembly. The analysis outputs included assembly QV scores and completeness statistics.

A Hi-C contact map was produced for the final, public version of the assembly. The Hi-C reads were aligned using bwa-mem2 ( Vasimuddin *et al.*, 2019) and the alignment files were combined using SAMtools ( Danecek *et al.*, 2021). The Hi-C alignments were converted into a contact map using BEDTools ( Quinlan & Hall, 2010) and the Cooler tool suite ( Abdennur & Mirny, 2020). The contact map is visualised in HiGlass ( Kerpedjiev *et al.*, 2018).

The final assembly was post-processed and evaluated using the blobtoolkit pipeline, a Nextflow port of the previous Snakemake Blobtoolkit pipeline ( Challis *et al.*, 2020). It aligns the PacBio reads in SAMtools and minimap2 ( Li, 2018) and generates coverage tracks for regions of fixed size. In parallel, it queries the GoaT database ( Challis *et al.*, 2023) to identify all matching BUSCO lineages to run BUSCO ( Manni *et al.*, 2021). For the three domain-level BUSCO lineages, the pipeline aligns the BUSCO genes to the UniProt Reference Proteomes database ( Bateman *et al.*, 2023) with DIAMOND ( Buchfink *et al.*, 2021) blastp. The genome is also split into chunks according to the density of the BUSCO genes from the closest taxonomic lineage, and each chunk is aligned to the UniProt Reference Proteomes database with DIAMOND blastx. Genome sequences without a hit are chunked with seqtk and aligned to the NT database with blastn ( Altschul *et al.*, 1990). The blobtools suite combines all these outputs into a blobdir for visualisation.

The genome evaluation pipelines were developed using nf-core tooling ( Ewels *et al.*, 2020) and MultiQC ( Ewels *et al.*, 2016), relying on the Conda package manager, the Bioconda initiative ( Grüning *et al.*, 2018), the Biocontainers infrastructure ( da Veiga Leprevost *et al.*, 2017), as well as the Docker ( Merkel, 2014) and Singularity ( Kurtzer *et al.*, 2017) containerisation solutions.

Table 4 contains a list of relevant software tool versions and sources.

**Table 4.** Software tools: versions and sources.

Software tool	Version	Source
BEDTools	2.30.0	https://github.com/arq5x/bedtools2
BLAST	2.14.0	ftp://ftp.ncbi.nlm.nih.gov/blast/executables/blast+/
BlobToolKit	4.3.7	https://github.com/blobtoolkit/blobtoolkit
BUSCO	5.4.3 and 5.5.0	https://gitlab.com/ezlab/busco
bwa-mem2	2.2.1	https://github.com/bwa-mem2/bwa-mem2
Cooler	0.8.11	https://github.com/open2c/cooler
DIAMOND	2.1.8	https://github.com/bbuchfink/diamond
fasta_windows	0.2.4	https://github.com/tolkit/fasta_windows
FastK	427104ea91c78c3b8b8b49f1a7d6bbeaa869ba1c	https://github.com/thegenemyers/FASTK
Gfastats	1.3.6	https://github.com/vgl-hub/gfastats
GoaT CLI	0.2.5	https://github.com/genomehubs/goat-cli
Hifiasm	0.19.8-r603	https://github.com/chhylp123/hifiasm
HiGlass	44086069ee7d4d3f6f3f0012569789ec138f42b84a a44357826c0b6753eb28de	https://github.com/higlass/higlass
Merqury.FK	d00d98157618f4e8d1a9190026b19b471055b22e	https://github.com/thegenemyers/MERQURY.FK
MitoHiFi	3	https://github.com/marcelauliano/MitoHiFi
MultiQC	1.14, 1.17, and 1.18	https://github.com/MultiQC/MultiQC
NCBI Datasets	15.12.0	https://github.com/ncbi/datasets
Nextflow	23.04.0-5857	https://github.com/nextflow-io/nextflow
PretextView	0.2	https://github.com/sanger-tol/PretextView
purge_dups	1.2.5	https://github.com/dfguan/purge_dups
samtools	1.16.1, 1.17, and 1.18	https://github.com/samtools/samtools
sanger-tol/ascc	-	https://github.com/sanger-tol/ascc
sanger-tol/ genomenote	1.2.2.	https://github.com/sanger-tol/genomenote
sanger-tol/ readmapping	1.2.1	https://github.com/sanger-tol/readmapping
Seqtk	1.3	https://github.com/lh3/seqtk
Singularity	3.9.0	https://github.com/sylabs/singularity
TreeVal	1.0.0	https://github.com/sanger-tol/treeval
YaHS	1.2a.2	https://github.com/c-zhou/yahs

### Wellcome Sanger Institute – Legal and Governance

The materials that have contributed to this genome note have been supplied by a Darwin Tree of Life Partner. The submission of materials by a Darwin Tree of Life Partner is subject to the **‘Darwin Tree of Life Project Sampling Code of Practice’**, which can be found in full on the Darwin Tree of Life website here. By agreeing with and signing up to the Sampling Code of Practice, the Darwin Tree of Life Partner agrees they will meet the legal and ethical requirements and standards set out within this document in respect of all samples acquired for, and supplied to, the Darwin Tree of Life Project.

Further, the Wellcome Sanger Institute employs a process whereby due diligence is carried out proportionate to the nature of the materials themselves, and the circumstances under which they have been/are to be collected and provided for use. The purpose of this is to address and mitigate any potential legal and/or ethical implications of receipt and use of the materials as part of the research project, and to ensure that in doing so we align with best practice wherever possible. The overarching areas of consideration are:

•   Ethical review of provenance and sourcing of the material

•   Legality of collection, transfer and use (national and international)

Each transfer of samples is further undertaken according to a Research Collaboration Agreement or Material Transfer Agreement entered into by the Darwin Tree of Life Partner, Genome Research Limited (operating as the Wellcome Sanger Institute), and in some circumstances other Darwin Tree of Life collaborators.

## Data Availability

European Nucleotide Archive: Vulpes vulpes (red fox). Accession number PRJEB74591; https://identifiers.org/ena.embl/PRJEB74591. The genome sequence is released openly for reuse. The *Vulpes vulpes* genome sequencing initiative is part of the Darwin Tree of Life (DToL) project. All raw sequence data and the assembly have been deposited in INSDC databases. The genome will be annotated using available RNA-Seq data and presented through the Ensembl pipeline at the European Bioinformatics Institute. Raw data and assembly accession identifiers are reported in Table 1 and Table 2.

## Author information

Members of the Natural History Museum Genome Acquisition Lab are listed here: https://doi.org/10.5281/zenodo.12159242.

Members of the Darwin Tree of Life Barcoding collective are listed here: https://doi.org/10.5281/zenodo.12158331.

Members of the Wellcome Sanger Institute Tree of Life Management, Samples and Laboratory team are listed here: https://doi.org/10.5281/zenodo.12162482.

Members of Wellcome Sanger Institute Scientific Operations: Sequencing Operations are listed here: https://doi.org/10.5281/zenodo.12165051.

Members of the Wellcome Sanger Institute Tree of Life Core Informatics team are listed here: https://doi.org/10.5281/zenodo.12160324.

Members of the Tree of Life Core Informatics collective are listed here: https://doi.org/10.5281/zenodo.12205391.

Members of the Darwin Tree of Life Consortium are listed here: https://doi.org/10.5281/zenodo.4783558.
