# Supplementary material for: The genome sequence of the red fox, Vulpes vulpes (Linnaeus, 1758)
Source: Wellcome Open Res. 2025 Jan 14;10:13. [Version 1] doi: 10.12688/wellcomeopenres.23516.1 (PMC11842965; doi:10.12688/wellcomeopenres.23516.1)
Supplement: Peer review history

# Reviewer report: Reviewer response for version 1

**DOI:** 10.21956/wellcomeopenres.25937.r118825

**Published:** 2025-03-07

**Authors:** Jonathan J Hughes

**Affiliations:** 1 University of California, Riverside, USA

This data note describes a new chromosome-level assembly for a Eurasian red fox – as previous reviewers have commented, this ancestry should be noted in the manuscript - which will be a valuable resource for studies of mammalian evolution. The methods and figures are clear and appear to follow best practices. Data is publicly archived, and all accession numbers appear to be correct. The authors should heed the suggestions of the three previous reviewers, which are sound. Additionally, the final paragraph of the background feels a bit flimsy; nothing in the preceding text indicates how analyses of this genome will help with informing public health strategies.

Are sufficient details of methods and materials provided to allow replication by others?

Yes

Is the rationale for creating the dataset(s) clearly described?

Partly

Are the datasets clearly presented in a useable and accessible format?

Yes

Are the protocols appropriate and is the work technically sound?

Yes

Reviewer Expertise:

Genomics, phylogenomics, mammalogy, evolutionary genetics

I confirm that I have read this submission and believe that I have an appropriate level of expertise to confirm that it is of an acceptable scientific standard.

# Reviewer report: Reviewer response for version 1

**DOI:** 10.21956/wellcomeopenres.25937.r118824

**Published:** 2025-03-07

**Authors:** Gabriela Pozo

**Affiliations:** 1 Universidad San Francisco de Quito, Quito, Ecuador

I congratulate the authors for this well-written manuscript and important results obtained. This article presents the sequencing and assembly of two haplotypes from a *Vulpes vulpes* individual, resulting in high-quality and highly complete genome assemblies. The methodology is well-detailed and clearly explained.

 While the rationale for generating these high-quality haplotypes is understandable, it would be beneficial to clarify how they enhance genomic analyses. Specifically, there is no mention of the existing reference genome, which is highly fragmented, nor how these new assemblies represent a significant improvement. I believe explicitly addressing this comparison would strengthen the manuscript.

Are sufficient details of methods and materials provided to allow replication by others?

Yes

Is the rationale for creating the dataset(s) clearly described?

Partly

Are the datasets clearly presented in a useable and accessible format?

Yes

Are the protocols appropriate and is the work technically sound?

Yes

Reviewer Expertise:

Genomics, Population Genetics

I confirm that I have read this submission and believe that I have an appropriate level of expertise to confirm that it is of an acceptable scientific standard.

# Reviewer report: Reviewer response for version 1

**DOI:** 10.21956/wellcomeopenres.25937.r117257

**Published:** 2025-03-07

**Authors:** Richard Edwards

**Affiliations:** 1 University of Western Australia, Perth, Australia; 2 School of Biotechnology and Biomolecular Sciences, University of New South Wales, Sydney, New South Wales, Australia

This manuscript describes a largely complete and contiguous phased genome assembly of a female (XX) red fox. The assembly data and methods are generally well described and are of high quality. However, there is a major omission with respect to the previously published red fox genome ( https://www.nature.com/articles/s41559-018-0611-6) and existing chromosome numbering. 

**Revisions/Comments**

 1. Existing fox genome. The previously published (draft) red fox genome (Kukekova et al. 2018, https://www.nature.com/articles/s41559-018-0611-6) needs to be acknowledged and cited. It would be useful to have a bit more rationale for generating another genome. Is this just about improving the quality, or is there more to differentiate this individual from the previous one? Given the availability of the species, it would also be good to explain the rationale of sequencing another female rather than a male, complete with Y chromosome. I would like to see some basic comparisons of this genome with the previous one, which was also scaffolded to chromosome level (see point 2, below). The draft genome was very fragmented, so comparisons should be favourable, but the original paper did feature a fair amount of analysis and so it would useful to know just how much of an improvement this is.

 2. Fox chromosomes. Looking at Fig 5, it appears that the chromosomes have been sorted by size and then numbered. The previous assembly used synteny mapping onto the dog genome to order and then number the dog chromosomes (Kukekova et al. 2018 Fig 2, https://www.nature.com/articles/s41559-018-0611-6). The fox karyotype seems to be established with existing numbering (e.g. Rubtsov 1998, https://academic.oup.com/ilarjournal/article/39/2-3/182/624271). I suspect the draft assembly matches this numbering, but it was not explicitly stated that I could see. To avoid future confusion, it is important to map the new reference genome onto the established chromosome numbers. Ideally, this would be accompanied with some synteny analysis against the draft genome to assess its scaffolding accuracy.

 3. Curation: “The assembly was decontaminated using the Assembly Screen for Cobionts and Contaminants (ASCC) pipeline (article in preparation).” This needs to be described or a link provided to published details, e.g. a pre-print or similar. If a link is unavailable, this should be a personal communication and name the authors, not “(article in preparation)”. 

 ( https://wellcomeopenresearch.org/for-authors/article-guidelines/preparing-a-data-note/#ref: “Unpublished abstracts, papers that have been submitted to a journal but not yet accepted, and personal communications should instead be included in the text; they should be referred to as ‘personal communications’ or ‘unpublished reports’ and the researchers involved should be named.”)

 4. Curation: “The process is documented at https://gitlab.com/wtsi-grit/rapid-curation (article in preparation).” This needs to link to a specific version/release or, if this is not possible (there do not appear to be versions or releases), provide the date of access and the github commit hash. “(article in preparation)” should be deleted.

Are sufficient details of methods and materials provided to allow replication by others?

Partly

Is the rationale for creating the dataset(s) clearly described?

Partly

Are the datasets clearly presented in a useable and accessible format?

Yes

Are the protocols appropriate and is the work technically sound?

Yes

Reviewer Expertise:

Genome assembly, bioinformatics, biodiversity genomics, conservation genomics, molecular evolution

I confirm that I have read this submission and believe that I have an appropriate level of expertise to confirm that it is of an acceptable scientific standard, however I have significant reservations, as outlined above.

## References

[1] : Red fox genome assembly identifies genomic regions associated with tame and aggressive behaviours. *Nat Ecol Evol* .2018;2(9) : 10.1038/s41559-018-0611-6 1479-1491 10.1038/s41559-018-0611-6 30082739 PMC6663081

[2] : A complete comparative chromosome map for the dog, red fox, and human and its integration with canine genetic maps. *Genomics* .1999;62(2) : 10.1006/geno.1999.5989 189-202 10.1006/geno.1999.5989 10610712

[3] : The Fox Gene Map. *ILAR J* .1998;39(2-3) : 10.1093/ilar.39.2-3.182 182-188 10.1093/ilar.39.2-3.182 11528077

# Reviewer report: Reviewer response for version 1

**DOI:** 10.21956/wellcomeopenres.25937.r117256

**Published:** 2025-02-27

**Authors:** Frank Hailer

**Affiliations:** 1 Cardiff University, Cardiff, Wales, UK

I agree with the assessments and suggestions by the two previous reviewers, who did a great job at picking up important and helpful points in my view.

 In addition, I would suggest the following addition, building on comments by reviewer 2: First sentence of second paragraph of Background section:  I suggest editing as follows: “Eurasian red foxes are found across a wide range of…urban areas, and range throughout Eurasia and North Africa (Statham et al. 2014), with Rüppell's fox mitochondrial DNA nested within the Palaearctic clade of red foxes (Basuony et al. 2023).”

Are sufficient details of methods and materials provided to allow replication by others?

Yes

Is the rationale for creating the dataset(s) clearly described?

Partly

Are the datasets clearly presented in a useable and accessible format?

Yes

Are the protocols appropriate and is the work technically sound?

Yes

Reviewer Expertise:

evolutionary and conservation genetics of mammals and birds

I confirm that I have read this submission and believe that I have an appropriate level of expertise to confirm that it is of an acceptable scientific standard.

## References

[1] : Paraphyly of the widespread generalist red fox (Vulpes vulpes ): introgression rather than recent divergence of the arid-adapted Rüppell’s fox (Vulpes rueppellii )?. *Biological Journal of the Linnean Society* .2023;138(4) : 10.1093/biolinnean/blad001 453-469 10.1093/biolinnean/blad001

# Reviewer report: Reviewer response for version 1

**DOI:** 10.21956/wellcomeopenres.25937.r118821

**Published:** 2025-02-20

**Authors:** Benjamin N. Sacks

**Affiliations:** 1 University of California Davis, Davis, California, USA

This manuscript describes a valuable resource for the study of mammalian evolution.  It was previously evaluated by an expert on the technical aspects of genome assembly and I have nothing to add to that facet.  My comments here pertain to the organism itself. I concur with the first reviewer that it is important to be explicit about the fact that the genome is of a Eurasian red fox, a species for which no reference genome has been previously assembled.  Historically, the North American red fox ( *Vulpes fulva*), for which a draft genome exists (Kukekova et al. 2018; Rando et al. 2018), was considered a distinct species from the Eurasian red fox (Desmarest 1820; Merriam 1900a,b). Although it was temporarily lumped with *Vulpes vulpes* (Churcher in 1959), it was again distinguished, this time based on phylogenomic evidence (Statham et al. 2014; Sacks et al. 2018).

 In addition to genetic analyses, which universally support the distinctiveness of Eurasian and North American red foxes, translocations that have resulted in the two continental forms co-mingling provide support the presence of some form of reproductive isolating mechanism.  For example, studies in Europe where fur-farm foxes of North American origin have escaped and established invasive populations, the two populations occur in sympatry yet apparently do not interbreed (Wierzbicki et al. 2021).  Although Eurasian and North American red foxes have been shown to hybridize on the eastern seaboard of the United States where Eurasian red foxes were introduced in the 1700s (Kaprowicz et al. 2014), a near complete lack of Eurasian Y chromosomes in the hybrid population contrasts with a moderate prevalence of Eurasian red fox mtDNA genomes (and autosomal admixture), suggesting some degree of male infertility in the F1 hybrids (Kuo et al. 2019). Thus, Eurasian and North American red fox genomes may harbor significant structural differences and warrant distinct assemblies.

 More minor issues were that some of the background material presented could be replaced with more accurate and fundamental information, and some of the literature citations selected for the background section could be replaced with more appropriate foundational references. 

 Specific editorial suggestions are:

 Title: Please insert “Eurasian”” (or “Afro-Eurasian,” if preferable) before “red fox

 First sentence of abstract: please insert “Eurasian” to say “We present a genome assembly from an individual female Eurasian *Vulpes vulpes *(red fox; Chordata, Mammalia, Carnivora, Canidae).”

 First sentence of Background section: Please insert “Eurasian” before “red fox” and replace “exhibiting …sexual dimorphism” with text about its relationship to the North American red fox. For example, “The Eurasian red fox ( *Vulpes vulpes*), the largest-bodied member of the genus, is most closely related to the North American red fox ( *Vulpes fulva*; Statham et al. 2014), sometimes considered to be the same species (Churcher 1959).”

 Last sentence, first paragraph of Background section: Please replace Couper 2016 with Lloyd 1981, which is a much better-known reference for the Eurasian red fox (full reference info below).

 First sentence of second paragraph of Background section:  I suggest editing as follows: “Eurasian red foxes are found across a wide range of…urban areas, and range throughout Eurasia and North Africa (Statham et al. 2014).”

 Third paragraph of Background section: I suggest deleting this entire paragraph and replacing with more fundamental information.  Most of this is applicable only to some populations of the species (e.g., breeding season). It is also arbitrary information about the species. Instead, I would add something like

 "As with other canids, Eurasian red foxes are territorial, typically monogamous, and both parents participate in rearing offspring (Lloyd 1981).  Although frequently trapped from the wild for their fur, Eurasian red foxes have not been used extensively in fur-farming, an industry that has derived its stock primarily from North American red foxes (Statham et al. 2011). Where such farmed foxes have escaped in Europe, they do not appear to interbreed with Eurasian red foxes (Wierzbicki et al. 2021).”

 First sentence under the header “Genome sequence report”: I realize you indicate this in the Methods, but for transparency, please specify here that the specimen originated in England, e.g., “The genome of an adult female *Vulpes vulpes* from England…”

 Table 1 under “Study title” please change to “ *Vulpes vulpes* (Eurasian red fox)”

 References: Please delete Couper 2016 and add relevant ones suggested above (full citations below).

Are sufficient details of methods and materials provided to allow replication by others?

Yes

Is the rationale for creating the dataset(s) clearly described?

Partly

Are the datasets clearly presented in a useable and accessible format?

Yes

Are the protocols appropriate and is the work technically sound?

Yes

Reviewer Expertise:

Canid genetics, genomics, evolution, ecology, and systematics

I confirm that I have read this submission and believe that I have an appropriate level of expertise to confirm that it is of an acceptable scientific standard.

## References

[1] : The Specific Status of the New World Red Fox. *Journal of Mammalogy* .1959;40(4) : 10.2307/1376267 10.2307/1376267

[2] Desmarest, A. G. 1820. Mammalogie, ou description des esp6ces de mammiferes, Vol. 1,p. 203. (In Encycl. Method.).

[3] : Fate of the other redcoat: remnants of colonial British foxes in the eastern United States. *Journal of Mammalogy* .2016;97(1) : 10.1093/jmammal/gyv179 298-309 10.1093/jmammal/gyv179

[4] : Red fox genome assembly identifies genomic regions associated with tame and aggressive behaviours. *Nat Ecol Evol* .2018;2(9) : 10.1038/s41559-018-0611-6 1479-1491 10.1038/s41559-018-0611-6 30082739 PMC6663081

[5] : Using Ancestry-Informative SNPs to Quantify Introgression of European Alleles into North American Red Foxes. *J Hered* .2019;110(7) : 10.1093/jhered/esz053 782-792 10.1093/jhered/esz053 31562767

[6] Merriam, C. H. 1900a. Preliminary revision of the North American red foxes. Proc. Wash. Acad. Sci., 2: 661-676.

[7] : Descriptions of twenty-six new mammals from Alaska and British North America.1900; 10.5962/bhl.title.46736 10.5962/bhl.title.46736

[8] : Construction of Red Fox Chromosomal Fragments from the Short-Read Genome Assembly. *Genes (Basel)* .2018;9(6) : 10.3390/genes9060308 10.3390/genes9060308 29925783 PMC6027122

[9] : Nuclear Genetic Analysis of the Red Fox Across its Trans-Pacific Range. *J Hered* .2018;109(5) : 10.1093/jhered/esy028 573-584 10.1093/jhered/esy028 29889225

[10] : Range-wide multilocus phylogeography of the red fox reveals ancient continental divergence, minimal genomic exchange and distinct demographic histories. *Mol Ecol* .2014;23(19) : 10.1111/mec.12898 4813-30 10.1111/mec.12898 25212210

[11] : Insight into the Genetic Population Structure of Wild Red Foxes in Poland Reveals Low Risk of Genetic Introgression from Escaped Farm Red Foxes. *Genes* .2021;12(5) : 10.3390/genes12050637 10.3390/genes12050637 PMC814607333922932

# Reviewer report: Reviewer response for version 1

**DOI:** 10.21956/wellcomeopenres.25937.r117250

**Published:** 2025-01-22

**Authors:** Ellie E Armstrong

**Affiliations:** 1 Stanford University, Stanford, California, USA

The Data Note from Colom and O’Brien presents a novel, chromosome-level assembly from a European red fox, which is highly contiguous. The data has been publicly archived on NCBI and is already available. The methods are mostly clear, but there are a few instances where I think additional clarity could be helpful. For instance, the paper refers to ‘combining the two haplotypes for curation’. It is not clear what this means and the context they were combined in. In addition, though the accession for the RNA sequencing data is provided, no use of the data is described. I would also recommend the section header for the Hi-C data method be modified to Hi-C and RNA library preparation and sequencing since this is also described here. Last, though I do not think it necessary to comment on this in a data note, I am surprised by the continuity of the contigs (2Mb) given the library stats from the Pacbio data. Usually, we see somewhere more in the range of 15-20Mb contig N50s from comparable assemblies. Perhaps I am overlooking something here? It is obviously more contiguous than the current red fox assembly on NCBI, but it is surprising to me nonetheless.

 My only other recommendation is that it may be significant to mention in the background section that the European fox is distinct from the North American red fox and that this sequence should represent the old-world lineage.

Are sufficient details of methods and materials provided to allow replication by others?

Partly

Is the rationale for creating the dataset(s) clearly described?

Yes

Are the datasets clearly presented in a useable and accessible format?

Yes

Are the protocols appropriate and is the work technically sound?

Yes

Reviewer Expertise:

Genome assembly, population genomics, evolutionary biology, conservation

I confirm that I have read this submission and believe that I have an appropriate level of expertise to confirm that it is of an acceptable scientific standard.
